# Continuity of care in children with special healthcare needs: a qualitative study of family’s perspectives

**DOI:** 10.1186/s13052-015-0114-x

**Published:** 2015-02-08

**Authors:** Elisa Zanello, Simona Calugi, Paola Rucci, Giulia Pieri, Silvia Vandini, Giacomo Faldella, Maria Pia Fantini

**Affiliations:** Department of Biomedical and Neuromotor Sciences – DIBINEM, Division of Hygiene and Biostatistics, Alma Mater Studiorum University of Bologna, Via San Giacomo 12-40126, Bologna, Italy; Neonatology and Neonatal Intensive Care Unit S.Orsola Malpighi Hospital, Department of Medical and Surgical Sciences, University of Bologna, Via Massarenti 11-40138, Bologna, Italy

**Keywords:** Continuity of care, Children with special health care needs, Hospitalization, Discharge, Community, Qualitative study, Empowerment, Patient engagement, Parents’ perspective, Interview

## Abstract

**Background:**

To explore parents’ experiences and perceptions on informational, management and relational continuity of care for children with special health care needs from hospitalization to the first months after discharge to the home.

**Methods:**

Semi-structured interviews and a focus group were carried out to capture parents’ experiences and perceptions. Transcripts were analyzed using a directed approach to the qualitative content analysis.

**Results:**

16 families participated to this study: 13 were involved in interviews (10 face-to-face and 3 by phone) and 3 in a focus group, within 1–6 months after discharge from the University Hospital of Bologna (S.Orsola/Malpighi) and from hospitals of Bologna Province. To parents of children with special health care needs, the three domains of continuity of care were relevant in a whole but with different key elements during hospitalization, at discharge and after discharge. Moreover, empowerment emerged from parents’ narratives as essential to help parents cope with the transition from the hospital setting to the new responsibilities connected with the home care of their child. Parent’s perceptions about the family pediatrician concerned his/her centrality in the activation and coordination of the healthcare network. Moreover, parents exhibited different attitudes towards involvement in decision making: some wished and expected to be involved, others preferred not to be involved.

**Conclusions:**

Care coordination for children with special care needs is a complex process that need to be attended to during the hospitalization phase and after discharge to the community. The findings of this study may contribute to elucidating the perceptions and experiences of parents with children with special health care needs about the continuity of care from hospital to community care.

**Electronic supplementary material:**

The online version of this article (doi:10.1186/s13052-015-0114-x) contains supplementary material, which is available to authorized users.

## Background

Children with special health care needs were defined by the Maternal and Child Health Bureau as those “who have a chronic physical, developmental, behavioral, or emotional conditions and who also require health and related services of a type or amount beyond that generally required by children” [1].

Although this population represents a category with a low prevalence and incidence, a large proportion of them require long-term treatments, including both inpatient and outpatient health care, with high economic impact on the healthcare system.

Medical care for children with special healthcare needs often requires a variety of services, providers and programs to implement complex care plans. Compared with adults, for children with complex chronic conditions the developmental status and the critical mediating role played by parents in the interaction between the child and the healthcare services and providers must be taken into account [2]. Strategies to connect patients, families, and providers with services and resources are needed to support coordinated, continuous care [3].

Continuity of care represents a key issue for children with special healthcare needs. According to the American Academy of Pediatrics, comprehensive healthcare should ensure *inter alia* continuity, providing care over an extended period of time and planning and organizing transitions, including those to other pediatric providers or into the adult health care system, with the child and family [4]. In the conceptualization developed by Reid and Haggerty, three types of continuity of care can be identified across healthcare settings, i.e. informational, management and relational [5,6].

However, to date little attention has been paid to the perspectives of families of children with special healthcare needs [7-9]. To our knowledge, only a qualitative study was conducted by Miller et al. [2] about continuity of care in which parents were interviewed to explore the extent to which their experiences and perceptions fit with the academic and service providers’ perspectives.

In Italy, information on prevalence of this condition and on services and resources activated for this population is scanty. Discharge procedures and care plans for children with special healthcare needs have been defined in some areas, with the aim to ensure comprehensive care and avoid the risk of fragmentation. In this context the family pediatrician plays an essential role as primary care provider for children up to 16 years of age and should ensure the coordination and continuity of care among healthcare services and providers. In Italy, where universal health care is provided, financed by the government through tax payments, pediatric primary health care is provided by family pediatricians that are remunerated on a capitation basis; they are in charge of providing care and assess patient’s needs, order diagnostic procedures, prescribe drugs, and refer patients to specialists and hospitals [10]. Thus, they act as ‘gatekeepers’ for the system.

Still, research supporting the content and use of care plans for children with chronic diseases and the family pediatrician’s role is limited as well as research about the family’s perspective lacks.

In Emilia-Romagna Region the SpeNK Project (Special Needs Kids) has been designed to describe the implementation of existing sheltered hospital discharge procedures and integrated clinical pathways for children with complex or chronic health conditions and special healthcare needs and to assess the family’s perspective on continuity of care and the role of family pediatrician.

Family perspectives on continuity of care have been explored with a qualitative research approach, referring to Reid and Haggerty’s constructs of informational, management and relational continuity of care [5,6] as conceptual basis, and using semi-structured methods. The aim of the present study is to examine the perceptions and experiences of families of children with special health care needs about these three constructs.

## Methods

### Study design

This qualitative study is part of the SpeNK Project. Children and families were recruited for SpeNK Project at hospital discharge of the child from the participating hospital facilities at the University Hospital of Bologna (S.Orsola/Malpighi) and the two local Health Authorities of Bologna and Imola. The recruitment of children was conducted from October 1st 2012 to September 30th 2014 on incident cases meeting the following inclusion criteria: age from 0 to 16 years, residence in Bologna province, and the presence of at least one of the following conditions:

- Birth weight <1000 g;
- Complex and/or chronic health conditions defined as:

- o Need for technological assistance,
- o Acute neurological deficit,
- o Severe endocrinopathy,
- o Complex genetic malformative pathology;

- Children with oncological diseases who need palliative care or particular community care;
- Newborns with mothers in contact with mental health services or drug addiction

Only first ever hospitalizations for the condition of interest were included.

Written informed consent was obtained at recruitment from each parent to collect clinical data on children and to contact them during follow-up period (9 months from hospital discharge).

We selected the first families recruited in SpeNK Project according to a maximum variation sampling method [11] with regard to the child’s diagnosis and the hospital of discharge and excluding parents with an inadequate level of knowledge of Italian language. We contacted one of the parents by phone and invited both, whenever possible, to participate in interviews or focus group.

### SpeNK-I interview and focus group

A semi-structured interview (SpeNK-I) was developed by the authors [see Additional file 1]. A selection of items, questions and probes was picked out from the international literature to explore a number of aspects of child care which are particularly relevant relevant in continuity of care [2,12]. Specifically, we examined parents’ experiences and perceptions about their child’s clinical condition and care plan (knowledge, communication, shared information and shared decision making), about service providers and clinicians involved in child’s care (who, how and why were involved, their availability and their information exchange) to explore relational and informational continuity, and about management continuity of care within the network of hospital and community service providers and clinicians.

The SpeNK-I was administered face-to-face or by phone at 1–6 months after discharge, lasted 60 minutes on average and was audiotape-recorded in the majority of cases. Where it was not possible (phone interviews), responses were documented in writing.

The SpeNK-I was then used to define the topics to be discussed in a focus group with parents, including (1) discharge, (2) coordination of care and overall organization of care provided both by hospital services and by community ones, (3) communication and shared information and decision making, (4) empowerment and proactive care received for their child’s care management during the hospital stay and after discharge.

The focus group lasted 150 minutes and was audiotape-recorded. All audio tapes were transcribed verbatim for analysis.

### Analysis

The semi-structured interviews were analyzed using a directed approach to the qualitative content analysis, as described by Hsieh and Shannon [13]. Qualitative content analysis is a research method for the subjective interpretation of the content of text data through the systematic classification process of coding and identifying themes or patterns [13]. In this study the directed approach to qualitative content analysis was chosen and used to validate or extend conceptually the theoretical framework of continuity of care.

The transcripts were read several times and all text related to the parents’ experiences and perceptions about the continuity of care was highlighted. Based on operational definitions of the three types of continuity of care [5,6], category codes were defined *a priori* and applied to the relevant text. *Informational continuity of care* addresses “the use of information on past events and personal circumstances to make current care appropriate for each individual” among providers and among healthcare events. *Management continuity of care* refers to “a consistent and coherent approach to the management of a health condition that is responsive to a patient’s changing needs”, which is especially important in chronic or clinically complex diseases. *Relational continuity of care* addresses “an ongoing therapeutic relationship between a patient and one or more providers”, which bridges past to current care and provides a link to future care [6].

All text that could not be coded within the predetermined coding scheme was identified and analyzed later for the attribution to a new category or a subcategory of an existing code. Therefore, some codes were developed inductively given their repeated appearance in the parents’ narratives. The entire process of reading and classification was conducted jointly by two investigators (one psychologist, EZ and one MD in Public Health, GP), and in case of disagreement the research team met to discuss the coding scheme and the attribution of issues to categories.

Ethical approval for the study was obtained from the University Hospital and Local Health Authorities’ Ethics Committees.

## Results

### Participants

Sixteen families (i.e. 15 mothers and 8 fathers) of 17 children participated in the study.

The characteristics of parents and of children are shown in Table 1.

**Table 1:** **Characteristics of participants (n = 16) and children (n = 17)**

Study participants (n = 16); n (%)	Mother only	8 (50.0%)
	Father only	1 (6.2%)
	Both parents	7 (43.8%)
Parental citizenship; n (%)	Both Italian parents	6 (37.5%)
	One Italian parent	3 (18.8%)
	No Italian parents	7 (43.7%)
Gender of children (n = 17); n (%)	Male	8 (47.1%)
	Female	9 (52.9%)
Age of children, months		Mean = 14.5, SD = 16.6; Median = 9.1; Range: 3.3-67.9
Time from discharge, months		Mean = 3.8, SD = 2.4; Median = 3.5; Range: 0.7-8.3
Children’s diagnosed health condition at discharge; n (%)	Prematurity < 1000 gr	9 (52.9%)
	Encephalopathy	5 (29.4%)
	Hydrocephalus	1 (5.9%)
	Myopathy	1 (5.9%)
	Malformation	1 (5.9%)
Hospital Unit; n (%)	Pediatric and Nursery Unit LHA Imola	2 (11.8%)
	Neonatology and NICU LHA Bologna	1 (5.9%)
	Pediatric Unit LHA Bologna	3 (17.6%)
	Pediatric Surgery Unit UH Bologna	3 (17.6%)
	Neonatology Unit UH Bologna	7 (41.2%)
	Pediatric Emergency Room UH Bologna	1 (5.9%)

Thirteen families were involved in interviews: most of them (10/13) were interviewed in their homes, while three were interviewed by phone at their request. The person interviewed was the mother in 6 cases, the father in one case and both in 6 cases. In 8 families at least one parent was immigrant. Three families (i.e. 3 mothers and 1 father) participated to the focus group and were represented by both parents in one case (both immigrant) and by the mother in the other two cases (Italian).

The interviews and the focus group were conducted at a median time of 3 months (range 1–11 months) from hospital discharge.

The median age of children was 7 months (range 1–68 months); 9/17 (52.9%) were preterm, 5/17 (29.4%) had a diagnosis of encephalopathy, 1/17 (5.9%) of hydrocephalus, 1/17 (5.9%) of myopathy, 1/17 (5.9%) of malformation.

11/17 (64.7%) children were recruited at discharge from the University Hospital of Bologna, 4/17 (23.5%) from the hospital of Bologna Local Health Authority and 2/17 (11.8%) from the hospital of Imola Local Health Authority (Table 1).

### Themes

The three categories of informational, relational, and management continuity of care, developed *a priori*, were confirmed by parents’ narratives. Moreover, the family empowerment was detected as new theme. Within these four major themes, three different phases were discernible (i.e. hospitalization, discharge, after discharge). Furthermore, two more themes, not *a priori* defined, were detected in parents’ narratives, referring to the role of family pediatrician and to the parents’ different attitudes about the wished level of involvement in decision making and information exchange.

**Figure 1:**
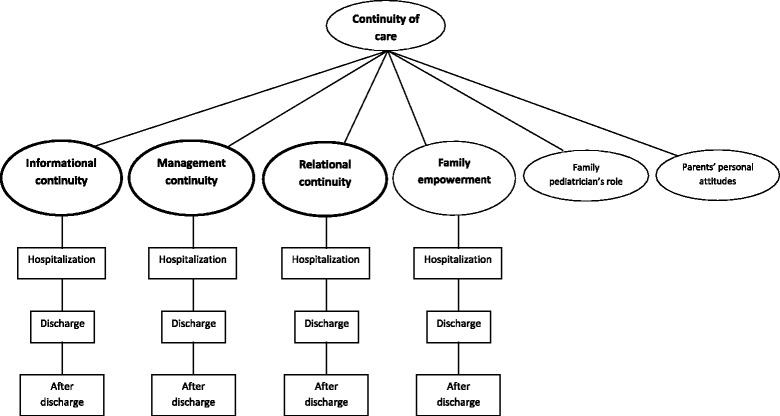
**Continuity of care for children with special health care needs in parents’ narratives.** The bold boxes represents the three *a priori* themes based on the literature, the other boxes represent the new emerged themes.

Figure 1 provides a visual representation of the themes and the phases aforementioned.

### Informational continuity

Communication and exchange of information represented critical functions of the interacting system of parents, child and clinicians, necessary for the informational continuity of care.

#### The hospitalization

Especially during the hospitalization, most of parents perceived the information exchange between them and clinicians and among clinicians or services as essential.

*“During hospitalization, information arrived or did not arrive. [Interview #13]*

*“There’s an efficient communication network, it works and sometimes it works too much: they know things before you do”. [Interview #08]*

*“If the parent, as it should be, is always or often there, he/she can help the nurse and vice versa”. [Interview #01]*

Explaining clinical and medical issues in an accessible way and with a comprehensible language to unskilled people, influenced significantly the understanding and learning possibilities of half of all parents about their child’s care.

*“The information is always… I mean … relative. You are completely unskilled and what you are told, are empty words, almost, they are not yet meaningful” “The recommendations are more prescriptive than descriptive” “The skilled people do not realize that, as unskilled person, you need to know the reason why you have to do something, because in that way, if nothing else, you internalize it” [Interview #08]*

#### The discharge

In few cases, the Discharge Letter filled by the hospital represented an essential information tool both for professionals and for parents in the transition from hospital to the home. For professionals it was a key element of the informational continuity of care because it ensured the information exchange among clinicians and services. For parents it represented a double-edged sword because it provided them with detailed medical information about the child’s hospitalization history, but was a source of concern if not well explained and understood.

*“What I didn’t like, was to find out many thing I didn’t know about my child from the discharge letter. It bothered us, and increased our fears, too”. [Interview #07]*

#### After discharge

After discharge, the parents’ experiences on the informational continuity of care in the healthcare pathway varied. In most cases, it was up to parents to ensure the informational continuity across health care settings and service providers, physically carrying medical reports, or orally communicating updates about child's conditions.

*“The letter of discharge… they gave it to us at the hospital, but none of the wards could access it (from the computer), so we always had to bring a printed copy for them” [Interview #02]*

### Management continuity

Elements of management (dis)continuity of care in parents’ narratives were related to the consistency of the care management by different providers and to the adaptation to child’s needs.

#### The hospitalization

During hospitalization, half of all parents perceived clearly the level of coherence and variability in the management of their child's care among hospital services and also within the same service staff.

*“The team of nurses (…) The team of doctors (…) They work the same way, in a team, and I liked it a lot” [Interview #06]*

*“They don’t have a common method, everyone does his/her own way [Interview #07].*

#### The discharge

The sheltered discharge procedure almost every time involved the family pediatrician, community services and parents in a meeting taking place in the hospital about a few days before discharge. It promoted the perception of being included in a care plan, within a network of health professionals, and in most cases ensured the continuity of care also by providing parents with all the contact details of the hospital, for any further need (information, doubts, worries, etc.).

*“We got all the contact information: they gave us a beautiful discharge folder, where you find all the numbers, contacts details of all the physicians of the Ward” [Interview #01]*

#### After discharge

The perception of management continuity was related in most cases to the presence or absence of a comprehensive care plan and in few cases also to the level of coherence between clinicians’ methods and protocols.

*“We were given a prospect of first steps to do, an agenda. (…) There's the follow-up of preterm newborns, then the pediatrician, the neurologist, the psychotherapist, they follow up us because we are under the kilo”. [Interview #07]*

*“The “post” (discharge) is a no man’s land” [FG]*

*“The problem is that everyone [every clinician] has his/her own way to do things. And now the community nurse has another method too (different from what we were used to during hospitalization)”. [Interview #02]*

### Relational continuity

Elements of relational continuity of care appeared in the parents’ narratives relating to the maintenance over time of an ongoing therapeutic relationship between the child and parents, and the health care providers.

#### The hospitalization

In the hospital setting, the continuity of the relationship between parents/child and care providers in the majority of cases related to the perception of familiarity, constant support and humanity of the professionals involved, essential for both children and parents.

*“The nurses are very sweet, kind, more than their job would require. They get to you heart… They do their work from the heart. There, they are all my child’s angels (…) They do not only ask me about my child, but also about me. They ask me: “how are you”?” [Interview #04]*

*“I had a good time there, with the head physician, the nurses, all the staff… the social health care operators were almost like family. After that, you consider them as relatives”. [Interview #11]*

#### The discharge

To a quarter of all parents, the transition from the hospital facility to home entailed a change in the relationships with professionals and service providers, and in the responsibilities connected to the child’s care.

*“I think that it is important to make parents feel that they’re are supported in the transition. We had the first meeting with all those professionals and we thought: “That’s great! Finally, they will really accompany us all the way”. Then, that’s not true. Probably the organization of the health care system doesn’t allow either a real communication or a well organized network making families feel supported, especially families like ours, living so far away” [Interview #08]*

*“All the mothers going home, after being many months at the hospital feel bad and scared. At home you’re 100% responsible: if something happens, what do I do?” [Interview #07]*

#### After discharge

When the network of hospital and community professionals works, it has an important function of reference point after discharge in most cases, for the follow-up visits and for any doubts or worries.

*“I always had the perception of being followed up (…) I always knew to whom I had to refer for specific problems”. [Interview #11]*

### Family empowerment

In the narratives of all the parents, the “empowerment” emerged as characterizing their experiences and perceptions in the interaction with the health care system. The family empowerment appeared as a process aiming to raise the parents’ ability to care their children, started during hospitalization, with the information and training provided by professionals.

#### The hospitalization

Half of all parents reported that during hospitalization they were involved in a specific training to manage every day and special needs of their child. The parents acknowledged the importance of being provided with adequate training and information, not only for the care activities but also for the relationship with their child.

*“In the hospital you learn (…) They teach you a lot of things, so when you are at home, you can manage you baby” [Interview #05]*

#### The discharge

In the experiences of one third of parents, the discharge appeared well planned and organized, with a specific focus on their training about their child’s care management at home.

*“From 20 days prior the hospital discharge, the nurses started to train us on practices and operations to do on the child with the medical devices” [Interview #02]*

#### After discharge

The empowerment of parents continued in half of cases involving them personally in the child’s care (e.g. daily physiotherapy exercises) after discharge.

*“With the physiotherapy, we are all involved (…) The large part (of rehabilitation activity) is done by us (…) The training she does in one hour of physiotherapy, doesn’t stop there”. [Interview #08]*

### The family pediatrician’s role

The role played by the family pediatrician raised mixed perceptions in parents. In few cases the pediatrician was a rarely seen and contacted person. In most cases, he/she played a pivotal mediating and coordinating role, activating the healthcare network or taking into account clinical aspects important to the parents and not included in the care plan.

*“The (family) pediatrician really made me smile. Before the hospitalization we didn’t see her. Now she makes home visits. In the previous 4 years, where was she?” [Interview #09]*

*“The (family) pediatrician is very present; whenever we need her, we call and she comes. (…)”. [Interview #12]*

*“The family pediatrician does not coordinate our child’s care. We coordinate everything […]. It. We prefer to do so. We want to care for our child”. [Interview #12]*

### Parents’ personal attitudes

In all cases, parents perceived differently the elements of (dis)continuity of care, based on their preferences about the wished level of information and involvement in decision making, during hospitalization, or of coordination in the healthcare management of their children, after discharge.

*“We are not physician, but we want do make decisions.” [Interview #12]*

*We didn’t ask much because we didn’t know what to ask” [Interview #03]*

## Discussion

This study is the first Italian study to explore parents’ perspective on the continuity of care for children with special health care needs during hospital stay, at discharge and in the first months after discharge.

Our results suggest that continuity of care issues varies from experiences related to interactions with a single professional and service during hospitalization to a global perception of being included in a comprehensive care plan within an integrated network of healthcare professionals and services, at discharge and after discharge.

Informational continuity during hospitalization concerns, in the majority of cases, the information exchange among parents, professionals and services. Compared to the after discharge experience, the hospital admission and stay emerged as the dominant theme in parents’ narratives, probably because it represents a tough experience, involving the whole family and the sick child. The hospitalization is an event characterized by high uncertainty, low predictability and high level of distress and emotional burden for families [14-16]. We found that the communication skills of professionals are particularly relevant in determining the outcome of the information flow in such situation. In contrast, the parents’ perceptions and experiences about the interaction and information exchange with the healthcare system at discharge and after discharge referred mainly to the availability of written documents for sharing medical information and to their commitment in transferring the information.

The relational continuity is expressed as a perception of familiarity and support, mainly during the hospitalization, when it refers to the “human” quality of relationships with the staff, whereas at discharge and after discharge it refers to the activation of a network of service providers and clinicians as reference points for the family and the child’s care.

The management continuity was perceived in terms of coherence and adaptability of the care provided to the child by multiple professionals and services within the hospital facility, whereas at discharge and after discharge referred mainly to the definition of a shared care plan with different professionals. Miller and colleagues found that compartmentalization (i.e. management discontinuity) was more likely among teams working in different settings and service sectors in parents’ narratives about the children with complex chronic health conditions [2]. On the contrary, we found that elements of management discontinuity of care occur also within the same healthcare setting, and this can be due to the lack of communication among the staff members.

These three conceptual categories of continuity of care in adult patients with chronic disease proved to be useful to describe parents’ experiences of children with special healthcare needs. However, several conceptual overlaps can be found in the narratives of parents and in many cases informational, relational and management continuity are not easily discernible and sometimes redundant. This indicates that the continuity of care is a complex theoretical construct that requires further investigation through qualitative and quantitative studies.

Concerning parents’ education and training about their own child’s care, we found that the empowerment process aiming to provide specific skills to manage the child both during hospitalization and at home is another theme extremely relevant from parents’ perspective. These particular activities should be scheduled and standardized with the special commitment of a component of the staff. When scheduled, this process was started by the hospital professionals shortly before the discharge and supported by the community professionals after discharge. These findings indicated that parents’ empowerment is an important issue when dealing with special healthcare needs children, throughout the entire process of care, form hospital to home. Special efforts should be made to achieve an effective alliance with parents and families in order to sustain and ameliorate children care.

In our study we also found two other relevant themes about the continuity of care: family pediatrician’s role and parents’ personal attitudes.

The role of family pediatricians varied a lot in the ways and in the extent of their participation in the child’s care after discharge, from a key person with a pivotal mediating and coordinating role in a network of clinicians and service providers, to a rarely seen and contacted person. These perceived differences could suggest a lack of clarity about the role of family pediatrician in ensuring the continuity of care for children with special healthcare needs. However, these differences could be ascribed to a different level of preparation or familiarity of the family pediatrician to care for children with various conditions. To our knowledge, only one study, investigating the willingness and ability of pediatricians to accept children and youth with special healthcare needs into their practices, was conducted in the U.S. by Agrawal and colleagues [17]. The results indicated that pediatricians do not feel prepared to care for all types of conditions and this problem reduces the ability to implement effective medical home care.

Our findings also indicated that parents reported different perceptions and experiences of continuity of care according to their preferences, attitudes and behaviors. In general, we found that the perception of a positive outcome or improvement of the child’s condition affected significantly parents’ satisfaction and appeared to reduce their willingness to express any criticism and to identify any unmet need. Furthermore, parents reported different preferences about the level of information and shared decision making especially during hospitalization, whereby someone wished to be asked for, involved and informed about any treatment provided to the child, someone else felt to have neither the competences nor the role to participate in decision making, and felt not to be able to understand medical issues. This specific finding suggests that, as for other medical fields (i.e. oncology), the willingness and capability to be informed and involved should be tested [18,19]. The engagement of the patient as care partner was identified by Haggerty et al. [12] as an independent dimension emerging from the factor analysis of a generic measure of continuity of care in adult patients. In a study conducted by Stille et al. [20], parent partnership in communication and decision making about subspecialty referrals for children with special needs was endorsed both by parents and clinicians, “though relatively greater enthusiasm from parents may signal the need for work in implementing this partnership”. The use of a care plan could be helpful to support parent engagement and build a partnership between parents and clinicians.

The literature about the overall parental experience during children hospitalization reports themes and issues similar to those emerged from our study. Interviewing parents with children admitted to 8 PICUs (Pediatric Intensive Care Units) in university medical centers in the Netherlands, Latour et al. [14] identified some major themes, most of which recurring also in our interviews: coordination of care, information management, parent participation, attitude of professionals, emotional intensity. Another qualitative study in a tertiary care Canadian university affiliated hospital’s PICU identified three dimensions of the parental role perceived by parents, including being present and participating in the child’s care, forming a partnership of trust with the PICU health care team, and being informed of the child’s progress and treatment plan [15]. Similarly to our findings, significant themes including the vividness of parents’ memories of admission, the intensity of distress associated with times of transition and the lasting impact of the experience were reported by Colville et al. [16] in a study assessing the impact on parents of a child’s admission to intensive care in an English teaching hospital’s PICU.

Similarly, a systematic review by Cleveland [21] identified the following six needs for parents with an infant in the Neonatal Intensive Care Unit (NICU): (a) accurate information and inclusion in the infant’s care, (b) vigilant watching-over and protecting the infant, (c) contact with the infant, (d) being positively perceived by the nursery staff, (e) individualized care, (f) a therapeutic relationship with the nursing staff. Moreover, four nursing behaviors were identified as meeting parents’ needs: (a) emotional support, (b) parent empowerment, (c) a welcoming environment with supportive unit policies, and (d) parent education with an opportunity to practice new skills through guided participation [21].

Given these evidences, we can conclude that parents’ experiences and perceptions about intensive care admission of their children are similar across different geographical, cultural, and organizational contexts.

### Limitations

Our results should be interpreted keeping in mind some limitations. First, this study reflected the experiences of a small number of participants, all from the same district (i.e. Bologna province) though referring to three different hospital facilities of discharge (i.e. University Hospital of Bologna, Bologna Local Health Authority hospital, Imola Local Health Authority hospital). This may limit the generalizability of findings to other contexts with different service organizations in other healthcare systems.

Second, this study excluded non-Italian speaking parents. Compared to Italian speaking parents, they could have different expectations and report different experiences about the interaction with the healthcare system, related to different cultural backgrounds and to difficulties in the language comprehension.

## Conclusions

In summary, the findings of this study suggest that a continuous and coordinated care should be targeted to the treatment phase (hospital vs. community), to take into account children’s changing needs. Moreover, the information provided and shared decision making in the healthcare services should be personalized according to the preferences of patients/families. The development of easy-to-use instruments measuring the preferred level of engagement could help to improve the quality of healthcare services.

The findings of this study contribute to deeper understanding the complexity of perceptions and experiences of parents with children with special health care needs about the continuity of care from hospital admission to home care, given the sparse available evidence on these themes. In particular, these findings may provide knowledge to clinicians and providers working with special health care needs children, and to policy makers in order to redesign services and to improve the quality of the care provided. The involvement of patients as co-designer of healthcare services has been recently promoted by the NHS Institute for Innovation and Improvement in the UK using the “Experience Based Design” approach, a new way of bringing patients and staff together to share the role of improving care and re-designing services [22].

Further research is needed to examine the generalizability and transferability of our results to clinical practice and to deeper understand the role that the family pediatrician should play in coordinating and ensuring the continuity of care for children with special health care needs. Moreover, the issues raised by this study may provide the background for developing self-report instruments to assess continuity of care for children with special health care needs from parents’ perspective, in order to improve and promote family-centered care.

## Additional file

Additional file 1:
**SpeNK-I Interview Guide - Parents’ perspectives about continuity of care for children with special health care needs.**
