# Supplementary material for: Continuity of care in children with special healthcare needs: a qualitative study of family’s perspectives
Source: Ital J Pediatr. 2015 Feb 8;41:7. doi: 10.1186/s13052-015-0114-x (PMC4328636; doi:10.1186/s13052-015-0114-x)
Supplement: Additional file 1: — SpeNK-I Interview Guide - Parents’ perspectives about continuity of care for children with special health care needs. [file 13052_2015_114_MOESM1_ESM.docx]

# SpeNK-I Interview Guide

## Parents’ perspectives about continuity of care for children with special health care needs

| DOMAIN | SUBTHEMES | SAMPLE QUESTIONS/PROBES |
| --- | --- | --- |
| INTRODUCTION | RESEARCHER | (…) |
|  | PROJECT AND INTERVIEW | (…) |
|  | OVERALL EXPERIENCE | It has been a while your child has been discharged from hospital. How did you feel at the time? Tell me about your experience. |
| CHILD’S CLINICAL CONDITION | KNOWLEDGE | When was it diagnosed? What does it involve? How does it impact family life? |
|  | COMMUNICATION | When/how/where were you told about it? Who told you?  Was it explained to you in a way that you could understand?  Did you have the chance to express you doubts? Did they answer?  Did they listen to your ideas/expectations? To your fears/concerns?  Did you feel “welcomed”? |
|  | SHARED  INFORMATION / DECISION MAKING | How did they explain the consequences on your child’s health?  Did they present options for your child’s care/treatments? Did they ask you to help make decisions? Did they show respect for what you had to say? |
| CARE PLAN | COMMUNICATION | Did they explain to you the care plan after discharge? In a way that you could understand?  Do you have any idea about what will happen now/in the future? Did they explain which clinicians/services will be involved in your child’s care?  If you had questions or worries, could you talk about it?  Could you express your ideas about your child’s care?  Did you feel “welcomed”? |
|  | SHARED  INFORMATION / DECISION MAKING | Did they present options for your child’s care plan? Did they ask you to help make decision? Did they show respect for what you had to say? |
|  | INVOLVED  SERVICE PROVIDERS AND CLINICIANS  who, how, why, availability | Which service providers and clinicians were involved in discharge?  Did they tell you they were available for you after discharge?  Did they tell you how to get more information?  Did you have any contact with the Pediatrician? Other Psychologist? |
| POST DISCHARGE | PROVIDED  CHILD-MANAGEMENT INFORMATION | Thinking about service providers and clinicians,  did they enable you to know  what to do your child’s daily treatments at home?  what to do to make your child’s health good or better?  how to cope with minor complications or accidents? |
|  | CLINICIANS AND SERVICE PROVIDERS NETWORK  case manager,  shared information and care  CONTINUITY OF CARE | Is there one service provider or clinician who is most involved with your child’s care? Which one? Who? Why?  Do you feel that clinicians/service providers share your child’s clinical information? That they agree about your child’s care plan?  Are there some services/clinicians that have been easier/ more difficult to access? Which ones? Why?  What do you think about continuity of care you’ve been receiving from this network of clinicians/service providers? |
| OPEN-ENDED QUESTION | OTHER IMPORTANT ITEMS | Is there anything that is important to you that I haven’t asked about? (episode, event, encounter) |
| CONCLUDING QUESTIONS | THANKS, CLARIFICATIONS, COMMENTARIES | Thank you for your time.  Do you have any question about this conversation?  Would you like to tell anything else about it? |
